# Measurement of polychlorinated biphenyls in different high consumption canned foods, using the QuEChERS/GC-MS method

**DOI:** 10.1016/j.fochx.2023.100957

**Published:** 2023-10-21

**Authors:** Faezeh Vali Mohammadi, Peyman Qajarbeygi, Nabi Shariatifar, Razzagh Mahmoudi, Majid Arabameri

**Affiliations:** a Food Hygiene and Safety, School of Public Health, Qazvin University of Medical Sciences, Qazvin, Iran; b Health Products Safety Research Center, Qazvin University of Medical Sciences, Qazvin, Iran; c Department of Environmental Health Engineering, School of Public Health, Tehran University of Medical Sciences, Tehran, Iran; d Food Safety Research Center (salt), Semnan University of Medical Sciences, Semnan, Iran

**Keywords:** Canned foods, GC-MS, Health risk assessment, Polychlorinated biphenyls, QuEChERS

## Abstract

Polychlorinated biphenyls (PCBs) are organic pollutants containing chlorine, which can be carcinogenic to humans. The current research focused on the heart risk and determination of PCBs levels in canned foods using the modified QuEChERS (fast, easy, cheap, effective, resistant and safe) method and gas chromatograph/mass spectrometer (GC-MS) technique. In this study, LOD (limit of detection), LOQ (limit of quantification), and recovery ranged from 0.06 to 0.32, 0.18 to 1.07 ng/g, and 97.05 to 102.5 %, respectively. In canned foods, the highest median of PCBs was PCB 52 (0.27 ± 0.20 ng/g fat) and the lowest median were PCB 28 and 138 (not detected in samples). Also, the maximum median of PCB 28, 52, 101, 138, 153, and 180 were detected in eggplant samples (0.06 ng/g), haricot samples (0.49 ng/g), eggplant samples (0.36 ng/g), eggplant samples (0.19 ng/g), eggplant samples (0.11 ng/g) and lentiform samples (0.66 ng/g), respectively. The median PCBs levels of oral exposure were estimated to be in the range of 9.80E-07to 4.30E-05 ng/g.d for all population groups, which were meaningfully lesser than the Tolerable daily intake value. The Monte Carlo simulation (MCS) outcomes indicated that the rank order of PCBs in adults based on incremental lifetime cancer risk (ILCR) was Lentiform (7.05E-8) > canned fish (5.73E-8) > Eggplant (5.38E-8) > Haricot (4.33E-8) > pasta source (2.06E-8); and in children was Lentiform (3.40E-7) > canned fish (2.72E-7) > Eggplant (2.44E-7) > Haricot (2.06E-7) > pasta source (9.83E-8). The median values of the ILCR induced oral exposure for all groups were within safe limits (lower than 10^−6^). The heat map and multivariate principal component analysis (PCA) showed significantly different contributions of PCBs profile in samples as the PCA axis scores were correlated with the type of cans. Based on the obtained outcomes, it can be concluded that the PCBs of canned food do not potential health risks to Iranian consumers.

## Highlights

• PCBs were investigated in canned foods samples using GC-MS technique.
• Health risk assessment was calculated according to per capita Iranian consumption.
• In all samples, the amount of PCBs was lower than the standard.
• Canned food does not potential health risks to Iranian consumers.

## 1 Introduction

Polichlorinated biphenyls (PCBs) is a group of compounds that belongs to this family of pollutants (Y. Kang et al., 2020). This group of includes 209 chemical compounds, which are divided into two main groups: dioxin-like PCBs and non-dioxin-like PCBs (Biljana, 2008; B. Škrbić, 2007; B. Škrbić & Đurišić-Mladenović, 2007). Although polychlorinated biphenyls consists of 209 congeners (Ahmadloo et al., 2019), only 150 of them are used in industrial consumptions (Costabeber et al., 2018, Fürst et al., 1992, Kim et al., 2004, Lorenzi et al., 2020).

Various sources that cause the accumulation of dioxins and PCBs in the environs include woodland fires, pesticides, oil factories, trucks, dyes and volcanic outbursts (Saktrakulkla et al., 2020). But it can be stated that the main and biggest sources of emission of these compounds are the burning of household waste, waste of municipal, waste of medical, fire in landfills, and agricultural and fires of forest (Stafilov et al., 2011).

Consequently, PCBs evaporate and enter the air, and this pollution can be deposited on plants and soil, and can also contaminate agricultural crops and animal fodder, and then be transferred to livestock (B. D. Škrbić & Marinković, 2019; B. D. Škrbić, Marinković, Antić, & Gegić, 2017).

PCBs and dioxins may enter the body (human) through skin absorption, ingestion, and inhalation. PCBs and dioxin compounds are stable in terms of stability in the environment and biological conditions, and when humans are exposed to these compounds for a long time, they can cause chronic poisoning (Jirdehi, Qajarbeygi, Hosseini, & Babaei, 2014). Through the atmosphere, these compounds can be deposited on the cover of leafy plants and on the soil, which acts as a natural sink (Adekunte et al., 2010, Schecter et al., 2006).

The presence of any PCB congener in any food item has implications for human health because each PCB congener has specific physicochemical properties that affect bioaccumulation, bioavailability, and toxicities. PCBs cause adverse human health effects by disrupting immune, reproductive, nervous, and endocrine systems (van den Berg et al., 2017). The IARC (International Agency for Research on Cancer) and World Health Organization (WHO) have categorized PCBs as 2A class, probable carcinogenic congeners (Comai, 2020).

Canned foods are a staple of many people's diets in today's world. A 2013 survey of more than 1,000 Americans found that more than 60 percent of respondents stated eating canned foods at least once or twice a week. As mentioned before, the consumption of canned food is increasing for several reasons. More precisely, the per capita consumption of canned food in the world is 1.1/can/week or 22.6 kg/person/year and per capita consumption of canned fish in the world is 2.2 kg/person/year (Comerford, 2015). In general, canned foods include canned meats such as tuna, canned vegetables such as beans, and canned mixes such as stews. According to the research that was mentioned earlier, polychlorinated biphenyl compounds are present in all sources of canned food like vegetable, animal.

Nowadays, several equipment are applied to identify PCB compounds in foodstuff, like gas chromatography–mass spectrometry (GC-MS), gas chromatography – electron capture detector (GC-ECD), gas chromatography coupled with high-resolution mass spectrometry (GC-HRMS) and gas chromatography-triple-quadrupole mass spectrometry (GC-QqQ-MS/MS). Among them, GC/MS is cheaper, easier, simpler and more widely used (Reddy et al., 2019, Yaminifar et al., 2021). To extract PCB compounds and to assess in foodstuff, a procedure was announced in 2003, which was quick, easy, cheap, effective, rugged, and safe, called the QuEChERS procedure (Chen et al., 2022, Özdemir et al., 2019, Rutkowska et al., 2018).

Human health risk assessment is employed to describe potential dietary exposure risks to obtain better comprehensive and reliable forecasts of risk factors and uncertainties (Khalili et al., 2022, Samiee et al., 2020, Shariatifar et al., 2020). Several researches newly have applied MCS to assess the improbability in human health risk assessment data as an accurate approach (Eghbaljoo-Gharehgheshlaghi et al., 2020, Karimi et al., 2021, Rezaei et al., 2021).

In recent years, there have been several researches in the measurement of fish, or other plant foods in the field of measuring these compounds (Biljana, 2008; B. Škrbić, 2007; B. Škrbić & Đurišić-Mladenović, 2007), but so far, these stable compounds have not been measured in canned food.

Considering the pollution of the food chain with PCBs and the increasing trend of canned food consumption, there is no comprehensive research on the measurement of PCBs in canned foods in Iran. Therefore, for the first time in Iran, the present research was conducted to measure polychlorinated biphenyls in various widely consumed canned goods using the QuEChERS/GC-MS method and to assess the health risk.

## 2 Material and method

### 2.1 Chemical and reagents

NaCl (Sodium chloride), ACN (acetonitrile), MgSO_4_ (magnesium sulfate), primary secondary amine (PSA), toluene, methanol, C18 and standard of analytical (PCB29) containing 2,4,5-trichlorobiphenyl were gotten from Merck (Darmstadt, Germany).

### 2.2 Collection of sample

Forty five canned food samples were analyzed. These canned foods were purchased from markets of Tehran, Iran, and transferred to the laboratory (June to July 2022).

At first, the statistics of the most consumed canned food in each vegetable, meat and mixed group were obtained from the Food and Drug Organization of Tehran city. The most consumed canned meat was tuna (fish meat, vegetable oil and table salt) and pasta (tomato sauce, soy bean, spice, table salt, vegetable oil and mushroom) vegetable canned including beans (beans, table salt, tomato sauce, vegetable oil) and lentils (lentils, table salt, tomato sauce, vegetable oil) and mixed canned including eggplant stew (eggplant, pepper, table salt, tomato sauce, vegetable oil) and other stews.

### 2.3 Sample preparation

The canned food samples were ready by a QuEChERS modified method (Yaminifar et al., 2021). First, 5 g of sample was regimented and poured into a 50 mL glass tube, and then 10 μL of PCB29 (internal standard) with a 10 ng/mL concentration and 20 mL of acetonitrile were added and shaken (5 min).

In the next stage, 6 mg of miscellaneous powder of MgSO_4_ and NaCl, with 4 to 1 ratio was added to the tube of glass and then it was vigorously shaken by hand and shaker and centrifuged at 5000 rpm for ten minutes.

Afterwards, 10 mL of the overhead solution was poured into a glass vial (15 mL) and the tube (glass) was placed in the freezer at −20 °C for fifteen minutes.

Subsequently, primary secondary amine (400 mg), C18 (40 mg), and 1 mL of toluene were added to the vial (glass).

Finally, it was poured into a tube (glass) and shaken for thirty seconds with a shaker and kept stable for 5 min and 1 μL was poured into the GC-MS apparatus (Rutkowska et al., 2018).

### 2.4 Conditions of instrumental analytical

For the measurement of PCB analytes, a GC equipment (model of 6890; Agilent Technologies, Palo Alto, CA, United States) with a mass quadrupole spectrometer (model of 5973) was applied. The GC-MS conditions were: the gas of carrier: helium (99.9 %); the mode of injection: splitless; the volume of injection: 2 µL; the temperature of injector: 290 °C; rate of flow: 1 mL per minute. Ii this study, the type of column was capillary (HP5-MS; thickness of film: 0.25 µL; length of column: thirty meters; internal diameter: 0.25 mm). Also, the temperature program of oven: temperature of initial: 90 °C, isothermal: 2 min, rate of first: five centigrade degree/meters to 280 °C and isothermal: three minutes. Finally, the SIM (selected ion monitoring) mode, was applied for the detector at the 70 eV EI (electron ionization).

### 2.5 Methods quantification

The fundamental parameters for PCBs evaluates were validated: LOQ (limit of quantification), recovery percent, linearity, precision, accuracy and LOD (detection of limit). To the linearity of methods, by adding different levels of standard (0.10–40 ng/mL) on dissimilar days (in triplicate) were prepared 5 samples (in series) and then injected them (in duplicate) into the GC-MS equipment. Additionally, for each triplicate were prepared 2 samples of control (negative), which one of them was without matrix and the other was without sample. For each of the evaluated PCB analytes, the achieved data were applied to draw a curve of calibration. In this study, using the assessment of the RSD (relative standard deviation) percent, the precision was estimated, moreover identified as the CV (coefficient of variation), which was lower than eighteen percent, representing the method effectiveness for repeatability (intra-day) and for intermediate accuracy (inter-day). For repeatability, by the levels with eight nomogram per milliliters of each PCBs analytes, 5 samples (equally) were prepared under analysis. According to the proposed procedure, these samples (all on the same day) were assessed, from phase of extraction until investigation by the GC-MS equipment. For the procedure accuracy, the method of standard addition was applied, including adding different identified quantities of the standards (certified) of each PCB into the matrix (before the sample preparation). For each PCB analyte, by the standard addition (in triplicate) at levels of 20, 10, 1, 0.1, 0.05and 0 ng/mL were prepared 6 samples and the determined quantities were associated with the amounts added. Finally, by the procedure of average blank value were evaluated the LOD and LOQ parameters.

### 2.6 Probabilistic health risk assessment (MCS method)

The Monte Carlo simulation has been often used to evaluate potential risks to human health from exposure to contamination in food. The estimated chronic daily intake (EDI, mg/kg. day) of individual NDL-PCBs were calculated to evaluate the risks to human health for two groups (children and adult). The basic equation is as follows:

(1) $$\mathrm{EDI}=\frac{C\times ED\times EF\times IR}{\mathrm{BW}\times AT}$$

Here, C (NDL-PCBs concentration (ng/g)), ED (exposure duration, years), EF (exposure frequency, days/year), IR (ingestion rate, 1 g/day) (Kalantari, 2015), BW (reference body mass, the mean weight (children and adults) is between 15 and 70 kg, respectively) (Samiee, Fakhri, Sadighara, Arabameri, Rezaei, Nabizadeh, Shariatifar, & Mousavi Khaneghah, 2020), and AT (AT is the mean time (25550 days)((Shariatifar, Rezaei, Sani, Alimohammadi, & Arabameri, 2020).

The ILCR from exposure to PCBs congeners (a probable human carcinogen) computed by Eq. (2) (Yijin Kang et al., 2020):

(2) ILCR=EDI×SF

Where ILCR is incremental lifetime cancer risk, EDI is estimated chronic daily intake and SF is carcinogenic slope factor of oral intake PCBs by USEPA’s (United States Environmental Protection Agency) guidance (2 mg/Kg b.w. per day) (EPA, 2003). Also, the 95th percentile values of the risk were presented in order to distinguish significant risk. In this study, MCS (Crystal Ball v 11.1.2.4.600 software) was applied to calculate the probable carcinogenic risk and non-carcinogenic attributes (EDI and ILCR). This method was introduced by USEPA to analyze uncertainty for risk management.

### 2.7 Statistical analysis

Statistical Analysis descriptive statistics (minimum and maximum, mean and standard deviation) Pre-test and Post-test mean were taken and compared by SPSS version 20. The Kolmogorov–Smirnov test was used to determine the distribution of the study parameters. The Kruskal–Wallis test was applied to determine the significance between groups. The principal component analysis is a multivariate technique and is widely used in food contamination with the aim to find out the associations between contaminants and food products (Heydarieh et al., 2020; B. D. Škrbić, Marinković, & Spaić, 2020). To further analyze the relationship between the type and quantity of polychlorinated biphenyls in different canned foods, we performed a heat map analysis using Clustvis software (https://biit.cs.ut.ee/clustvis/) (Moradi et al., 2021).

## 3 Result and discussion

### 3.1 Characteristics of technique

Analytical characteristics of technique for the examination of PCB compounds revealed in Table 1. In this study, LOD, LOQ, recovery and RSD ranged from 0.06 to 0.32, 0.18 to 1.07 ng/g, 97.05 to 102.5 % and 5.66 to 9.50 %, respectively. Compared with our study, Fathabad et al. analyzed PCB compounds by using Soxhlet Extractor with HRGC/HRMS and expressed LOQ for all dl-PCBs ranged between 0.03 and 0.09 pg/g fat, also, in other study by Kiani et al. that used modified QuEChERS/GC-QqQ-MS/MS method, they expressed the LOQs, LODs, recovery, and RSD for the PCB analytes were 0.180–0.360, 0.06–0.12 ng/g fat, 97.45–102.63 %, and 6.33–8.86 %, respectively, furthermore, in other study by Shahsavari et al. that used modified QuEChERS extraction and GC-QqQ-MS/MS method they stated the LOD, LOQ and recovery for the PCB analytes were varied 0.04–0.16, 0.132–0.482 ng/g fat, 5.2–9.2 and 95.5–107.2 %, respectively, and finally in study of Shariatifar et al. that used modified QuEChERS extraction and GC-QqQ-MS/MS method, they stated the recovery, LOD and LOQ of NDL-PCBs were varied 93.22–109.19 %, 0.04 to 0.14 and 0.120 to 0.420 ng/g, respectively (Fathabad et al., 2020, Kiani et al., 2023, Shahsavari et al., 2022, Shariatifar et al., 2022).

**Table 1:** Characteristics of technique.

Compound	Linear range of concentration (ng/mL)	LOD (ng/g)	LOQ (ng/g)	RSD%	Recovery%	Intra-day	Inter-day
PCB 18	0.10 – 40	0.12	0.40	6.69	99.75	7.12	8.36
PCB 28	0.10 – 40	0.080	0.250	7.23	98.98	8.97	9.18
PCB 31	0.10 – 40	0.14	0.47	5.75	97.05	7.05	9.21
PCB 44	0.10 – 40	0.32	1.07	5.66	98.63	7.65	10.06
PCB 52	0.10 – 40	0.080	0.250	6.49	99.14	9.72	12.18
PCB 101	0.10 – 40	0.120	0.360	8.54	100.2	10.14	14.1
PCB 138	0.10 – 40	0.060	0.180	6.87	102.3	8.96	12.86
PCB 141	0.10 – 40	0.17	0.57	6.11	101.01	9.76	11.64
PCB 149	0.10 – 40	0.16	0.53	6.52	99.76	8.63	10.54
PCB 153	0.10 – 40	0.100	0.310	7.65	97.15	11.14	13.22
PCB 180	0.10 – 40	0.080	0.250	7.75	100.2	10.17	14.54
PCB 194	0.10 – 40	0.32	1.07	9.50	102.5	11.76	15.11

### 3.2 Assessment of PCBs in canned foods

The expressive statistical analysis of gained values, demonstrates the median, minimum and maximum of each analyte of PCBs (Table 2). The Kruskal Wallis test showed that PCB 18, PCB 28, PCB 44, and PCB 101 the concentration varies significantly in different types of canned foods (p < 0.05) (Table 2). The highest and the lowest amount of NDL-PCBs in canned fish, which is known as tuna, belongs to PCB 52 with amounts of 0.56 and 0.18 ng/g fat, respectively. Continuously, PCB 52 had the maximum amount of NDL-PCBs in comparison with other indicator PCBs, in pasta source (Max.:0.27, Min.: 0.00 ng/g fat), haricot (Max.:0.88, Min.: 0.10 ng/g fat) and lentiform (Max.:0.47, Min.: 0.43 ng/g fat). However in eggplant canned samples, PCB 101 was the compound with the highest amount (Max.:0.0.53, Min.: 0.24 ng/g fat).

**Table 2:** Assessment of concentration of PCBs among different types of canned foods (ng/g).

products		PCB 18	PCB 31	PCB 28	PCB 44	PCB 52	PCB 101	PCB 141	PCB 149	PCB 138	PCB 153	PCB 180	PCB 194
Canned fish	Minimum	0.01	0.00	0.00	0.00	0.18	0.00	0.00	0.00	0.00	0.00	0.00	0.00
	Maximum	0.15	0.06	0.18	0.47	0.56	0.26	0.06	0.18	0.09	0.16	0.3	0.75
	Median	0.09	0.00	0.01	0.02	0.34	0.24	0.05	0.03	0.00	0.06	0.15	0.58
	Std.Deviation	0.05	0.02	0.09	0.18	0.13	0.1	0.03	0.07	0.04	0.06	0.1	0.3
Pasta source	Minimum	0.07	0.00	0.00	0.01	0.00	0.13	0.00	0.04	0.00	0.01	0.04	0.1
	Maximum	0.12	0.00	0.00	0.18	0.27	0.26	0.13	0.16	0.05	0.1	0.18	0.19
	Median	0.09	0.00	0.00	0.1	0.22	0.16	0.00	0.08	0.00	0.06	0.11	0.12
	Std.Deviation	0.02	0.00	0.00	0.06	0.11	0.05	0.05	0.04	0.02	0.04	0.05	0.04
Haricot	Minimum	0.05	0.00	0.00	0.03	0.1	0.09	0.00	0.02	0.02	0.02	0.12	0.08
	Maximum	0.24	0.00	0.00	0.18	0.88	0.23	0.08	0.03	0.11	0.06	0.14	0.46
	Median	0.15	0.00	0.00	0.10	0.49	0.17	0.04	0.02	0.07	0.04	0.14	0.25
	Std.Deviation	0.11	0.00	0.00	0.08	0.44	0.08	0.05	0.00	0.05	0.02	0.01	0.21
Lentiform	Minimum	0.24	0.00	0.00	0.26	0.43	0.27	0.02	0.03	0.00	0.07	0.63	0.12
	Maximum	0.26	0.00	0.00	0.28	0.47	0.3	0.02	0.03	0.00	0.07	0.69	0.14
	Median	0.25	0.00	0.00	0.27	0.45	0.29	0.02	0.03	0.00	0.07	0.66	0.13
	Std.Deviation	0.02	0.00	0.00	0.02	0.03	0.02	0.00	0.00	0.00	0.00	0.04	0.01
Eggplant	Minimum	0.13	0.00	0.00	0	0.25	0.24	0.00	0.00	0.00	0.08	0.07	0.09
	Maximum	0.16	0.00	0.13	0.02	0.31	0.53	0.62	0.07	0.42	0.15	0.22	0.2
	Median	0.15	0.00	0.06	0.01	0.27	0.36	0.28	0.04	0.19	0.11	0.14	0.14
	Std.Deviation	0.02	0.00	0.07	0.01	0.03	0.15	0.34	0.04	0.23	0.03	0.08	0.05
Total	Number	45	45	45	45	45	45	45	45	45	45	45	45
	Minimum	0.01	0.00	0.00	0.00	0.00	0.00	0.00	0.00	0.00	0.00	0.00	0.00
	Maximum	0.26	0.06	0.18	0.47	0.88	0.53	0.62	0.18	0.42	0.16	0.69	0.75
	Median	0.1	0.00	0.00	0.08	0.27	0.23	0.02	0.04	0.00	0.06	0.14	0.16
	Std.Deviation	0.06	0.01	0.06	0.12	0.2	0.11	0.15	0.05	0.1	0.05	0.15	0.23

In this table, zero means less than LOD.

According to the mentioned standard in previous study (Commission Regulation (EU) NO1259/2011) the limit of acceptable for PCBs in herbaceous, meaty and mixed canned foods are 40 ng/g (EU, 2011, Yaminifar et al., 2021).

According to the outcomes of present study, the content of all considered polychlorinated biphenyls compounds in canned foods were lower than the limitations of standard. The outcomes displayed the mean levels of total NDL-PCBs in canned foods for PCB 28, 52, 101, 138, 153 and 180 was 0.06, 0.27, 0.36, 0.19, 0.11, 0.14 respectively, with a maximum concentration of 0.36 ng/g fat belonging to PCB 101. The PCB with the highest mean was PCB 52 (0.27 ± 0.20 ng/g fat) and the concentration of maximum was 0.88 ± 0.20 ng/g fat. The minimum average were PCB 28 and 138 (not detected).

In this study, a comparison with other studies is presented in Table 3.

**Table 3:** Comparison of the present study with other researches.

Researcher	Year	Compounds	Samples	Results	References
El Morsy et al.	2013	PCBs	Canned and tuna	21.35 to 55.1 ng/g and 8.56 to 208.11 ng/g, respectively	(El Morsy et al., 2013)
Tesi et al.	2020	PCBs	Canned sardines	<LOD to 668 ng/g	(Tesi & Iniaghe, 2020)
Kipčić & Vukušić	1991	PCBs	Canned fish	5.0 to 4175 ng/g (mean 194 ng/g)	(Kipčić & Vukušić, 1991).
JuanM et al.	2003	PCBs	Vegetables, fruits, cereals, fish and shellfish, meat and meat products, eggs, milk and dairy products, oils and fats	Mean of PCBs in fish and oysters was 18.118 ng/g, in milk and dairy products was 5.674 ng/g and in oils and fats was 53.451 ng/g.	(Llobet et al., 2003).
Baars et al.	2004	-PCDD, dl-PCBs and NDL-PCBs	Meat products, dairy products, fish, eggs, vegetable products and fats and oils	The average level of PCDD/Fs + dl-PCBs in vegetable oils is 0.18 pg/g and the mean level of indicator PCBs in these products was 1.3 ng/g.	(Baars et al., 2004).
Yagüe et al.	2005	pesticides and PCBs	Virgin olive oil	Only one organophosphate pesticide was identified in one sample, the level of which was 10 ng/g chlorine pesticides were also detected in 5–47 % of the samples in very low concentrations.	(Yagüe et al., 2005).
Adenugba, Headley, McMartin, & Beck	2008	PCBs	Vegetable oils and oil-containing products	The mean concentration of these pollutants was lower than the standard limit. The concentration of human exposure to PCBs in entire products was below 1 ng/g body weight per day, considering a 70 kg in men or 57 kg in women.	(Adenugba et al., 2008).
Ahmedloo et al.	2018	PCBs	Egg samples	amount of PCBs was lower than the standard and this case	(Ahmadloo et al., 2019)
Yaminifar et al.	2021	NDL-PCBs	Butter samples	the average level of NDL-PCBs in traditional butters, which was 21.701 ± 9.02 ng/g fat	(Yaminifar et al., 2021).
Biljana et al.	2008	NDL-PCBs	Edible sunflower oil, white sugar and wholegrain wheat flour	total NDL-PCBs was ranged 0.17 to 2.89 ng/g ww	(Biljana, 2008).

Generally, the amount of PCBs in present study, in comparison with the others, concluded that environmental contaminations of main sources like air, soil and water with pesticides, oil products, pollutants of trucks, paints and demolition of houses can lead to the agricultural crops and animals pollution. This is more serious when animal farms are near to roads or industrial sites. In general, the concentration of indicator PCBs including PCB 138,153,28, 180 and 52 in the canned foods were lesser than the EU standards (Adenugba et al., 2008, Baars et al., 2004, Llobet et al., 2003, Yagüe et al., 2005, Yaminifar et al., 2021).

### 3.3 Assessment of health risk

A health risk assessment was made according to the content of PCBs in different high-consumption canned foods. The predicted health risk of PCBs in the canned foods sold in the market of Iran is presented in Table 4.

**Table 4:** Analysis of uncertainty for the EDI of studied PCBs in canned food samples.

canned food		*adults*				*children*			
	**Percentiles**	5 %	50 %	75 %	95 %	5 %	50 %	75 %	95 %
*Eggplant*	PCB 18	1.4E-06	2.1E-06	2.5E-06	3.2E-06	6.6E-06	1.0E-05	1.2E-05	1.5E-05
	PCB 52	2.5E-06	3.8E-06	4.5E-06	5.8E-06	1.2E-05	1.8E-05	2.1E-05	2.6E-05
	PCB 101	3.4E-06	5.1E-06	5.9E-06	7.5E-06	1.6E-05	2.4E-05	2.8E-05	3.6E-05
	PCB 180	1.4E-06	2.0E-06	2.4E-06	3.0E-06	6.2E-06	9.1E-06	1.1E-05	1.4E-05
	PCB 194	1.3E-06	2.0E-06	2.3E-06	2.9E-06	6.2E-06	9.2E-06	1.1E-05	1.4E-05
*fish*	PCB 52	3.2E-06	4.8E-06	5.6E-06	7.3E-06	1.5E-05	2.2E-05	2.6E-05	3.3E-05
	PCB 101	2.3E-06	3.4E-06	3.9E-06	5.0E-06	1.1E-05	1.6E-05	1.9E-05	2.4E-05
	PCB 180	1.5E-06	2.2E-06	2.5E-06	3.2E-06	6.6E-06	9.8E-06	1.2E-05	1.5E-05
	PCB 194	5.5E-06	8.4E-06	9.6E-06	1.2E-05	2.6E-05	3.8E-05	4.5E-05	5.7E-05
*Haricot*	PCB 18	1.4E-06	2.1E-06	2.5E-06	3.2E-06	6.6E-06	1.0E-05	1.2E-05	1.5E-05
	PCB 52	4.6E-06	6.9E-06	8.1E-06	1.1E-05	2.2E-05	3.2E-05	3.8E-05	4.8E-05
	PCB 101	1.6E-06	2.4E-06	2.8E-06	3.5E-06	7.5E-06	1.1E-05	1.3E-05	1.7E-05
	PCB 138	6.6E-07	9.8E-07	1.2E-06	1.5E-06	3.0E-06	4.6E-06	5.4E-06	6.9E-06
	PCB 180	1.4E-06	2.0E-06	2.4E-06	3.0E-06	6.2E-06	9.1E-06	1.1E-05	1.4E-05
*Lentiform*	PCB 18	2.4E-06	3.5E-06	4.2E-06	5.4E-06	1.1E-05	1.7E-05	2.0E-05	2.5E-05
	PCB 52	4.2E-06	6.3E-06	7.5E-06	9.7E-06	2.0E-05	3.0E-05	3.5E-05	4.4E-05
	PCB 101	2.7E-06	4.1E-06	4.7E-06	6.0E-06	1.3E-05	1.9E-05	2.3E-05	2.9E-05
	PCB 180	6.4E-06	9.5E-06	1.1E-05	1.4E-05	2.9E-05	4.3E-05	5.2E-05	6.5E-05
*Pasta source*	PCB 52	2.1E-06	3.1E-06	3.7E-06	4.7E-06	9.7E-06	1.4E-05	1.7E-05	2.1E-05
	PCB 101	1.5E-06	2.3E-06	2.6E-06	3.3E-06	7.0E-06	1.1E-05	1.3E-05	1.6E-05
	PCB 180	1.1E-06	1.6E-06	1.9E-06	2.4E-06	4.9E-06	7.2E-06	8.6E-06	1.1E-05

**Fig. 1:**
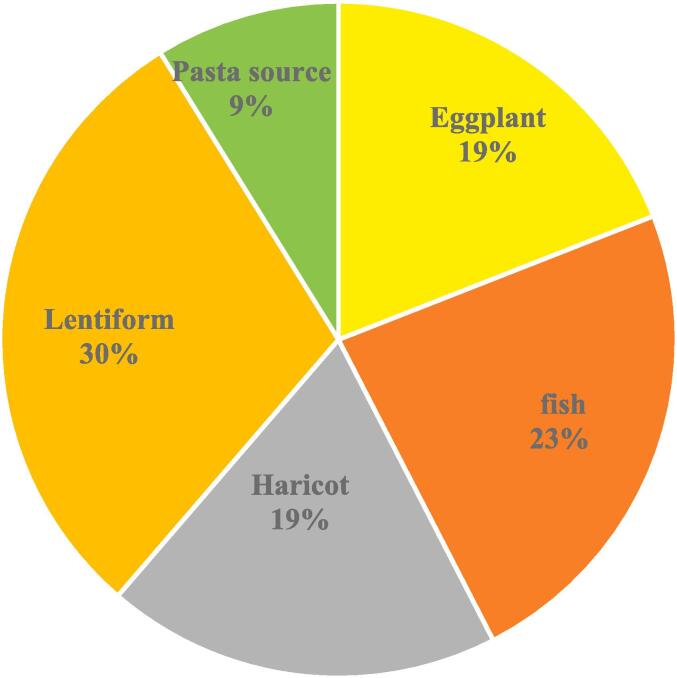
Comparison of the most and least contribution to overall EDI in canned samples.

Among all PCBs, health risk was found to be associated with PCB 18, PCB 52, PCB 101, PCB 141, PCB 138, PCB 180, and PCB 194 in the canned foods sold in the Iranian market. Van den Berg et al., (2017) founded that PCBs posed a risk to health of human even in moderate concentration, the continuous intake could cause health hazards in the long term (van den Berg et al., 2017). The predicted EDI of PCBs in the canned foods sold in the Iranian market is showed in Table 3. This outcomes displayed that the EDI values were below the current tolerable daily intake of PCBs (TDI < 10 ng kg^−1^ BW day^−1^). A higher EDI index was detected in the children. The contributions by concentration to EDIs were 30 and 23 % for lentiform and fish canned, respectively. The level of each congener pattern showed in Fig. 1. In lentiform, the highest quantity of EDIs was PCB 180, and the second was PCB 52.

In this way, the EDI of PCBs in some crop products was assessed to be 172.2 ng/kg per day for the population in Serbia (Biljana, 2008). Kang et al. (2020) founded that the PCBs in food samples in China and displayed the EDI of the ΣIndicator-PCBs was 26.47 ng/kg per day, which was upper than our research (Yijin Kang et al., 2020). While, Chung et al. (2018) stated that the lesser and higher bound exposures to indicator PCBs was 0.68 and 1.38 ng/kg day in the food samples, respectively (Chung, Lau, & Chu, 2018).

**Fig. 2:**
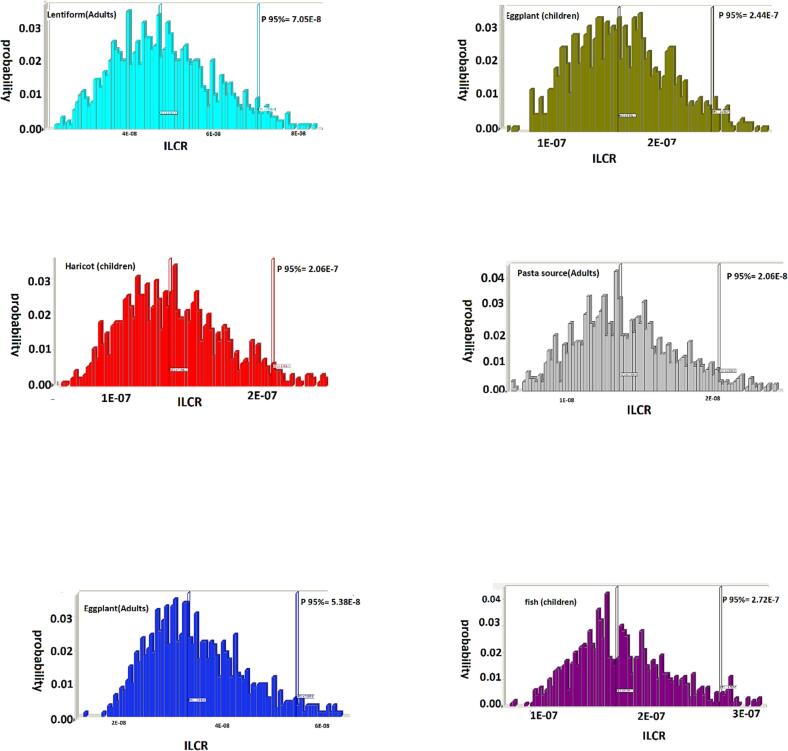

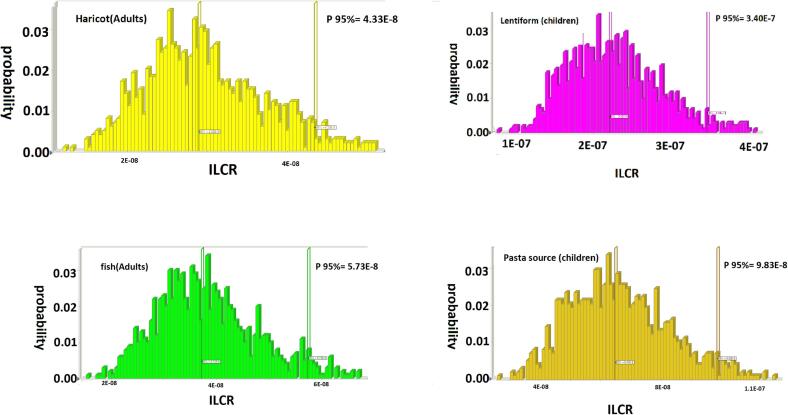
The results of simulation for EDI and ILCR of PCBs detected in canned samples.

A MCS method or multiple probability simulation was executed to quantitatively estimate the potential oral cancer risk for children and adults. Estimated carcinogenic and mutagenic risk values of PCBs in canned foods sold in the market of Iran are presented in Fig. 2. The rank order canned samples based on the ILCR index (95 %) in adults was Lentiform (7.05E-8) > canned fish (5.73E-8) > Eggplant (5.38E-8) > Haricot (4.33E-8) > pasta source (2.06E-8); and in children was Lentiform (3.40E-7) > canned fish (2.72E-7) > Eggplant (2.44E-7) > Haricot (2.06E-7) > pasta source (9.83E-8). The risk of cancer PCBs for the goal population was much lesser than the US EPA’s acceptable level of 10^−6^, indicating an acceptable cancer risk in consumer exposure to the PCBs. Regardless, this value is an issue to changes with an increase in exposure period and frequency. Given the potential value of risk assessment for addressing ILCR value, food products and exposure to PCB could be an important role in governing potential cancer risks in humans.

### 3.4 Analysis according to heat map outcomes

**Fig. 3:**
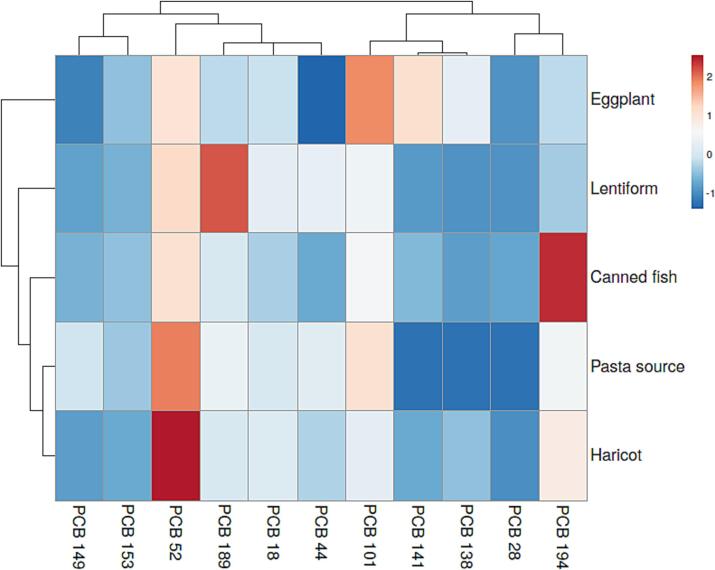
Heat map of PCBs in canned samples.

Heat maps by intuitive visualization of data and results can be useful to present a very brief view of a specific purpose. The hierarchical clustering analysis dendrogram of PCBs in the canned foods is presented in Fig. 3. As the Euclidean space reduced, the samples shows an upper correlation together. As can be observed, the samples were well divided into two main clusters that present the correlation between the amount and type of PCBs in canned food.

**Fig. 4:**
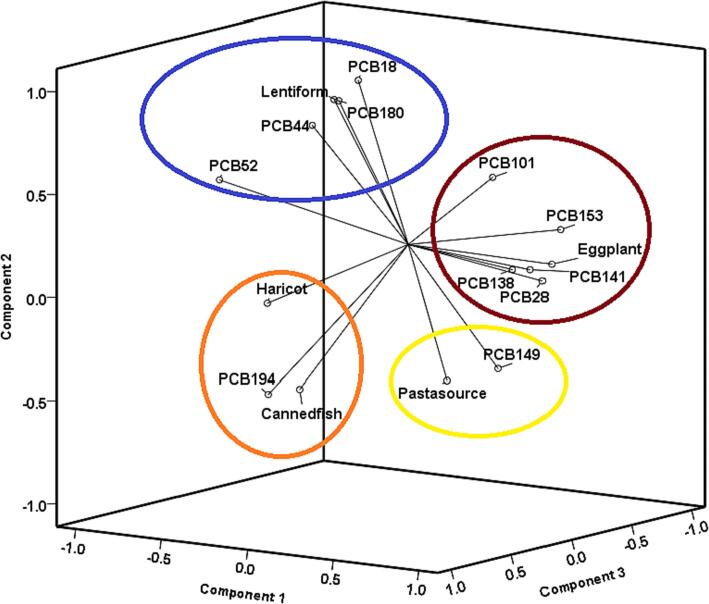
Principle Component Analysis (PCA) of PCBs in canned samples.

Different samples based on their contamination with PCB 138, PCB 28, PCB 194, PCB 101, and PCB 141 were clustered together in different type, as the first category. Hence, these PCBs had a highly similar trend in different samples (canned fish, pasta source, haricot, lentiform and eggplant). The other samples showed a great correlation among PCB 149, PCB 153, PCB 52, PCB 198, PCB 18, and PCB 44, as the second category. Also, the study canned sample consisted of two clusters, the first cluster only includes eggplant, and the second cluster includes canned fish, pasta source, haricot, and lentiform. The heat map provided a quantitative description of data sets between row and column clustering and sparse PCBs congeners with a high correlation degree. Principle Component Analysis (PCA) was performed in order to clarify the general distribution patterns or similarities of individual PCB in canned food (B. Škrbić, Szyrwińska, Đurišić-Mladenović, Nowicki, & Lulek, 2010). The PCA possessed a satisfactory sum of proper values with component 1, component 2 and component 3 axes (38.35 % for *P*1, 29.43 % for *P*2 and 19.483 for *P*3). The parameter plot (Fig. 4) was primarily structured by the component 1 axis positively characterized by the PCB 28, PCB 101, PCB 141, PCB 153, PCB 138 and eggplant (cos2 ≥ 0.5), and negatively by the PCB 44, and PCB 52 (cos2 ≤  − 0.5). Also, component 2 axis positively characterized by the PCB 52, PCB 18, and PCB 44 and canned lentils (cos2 ≥ 0.7), and negatively by the PCB 149 (cos2 ≤  − 0.5). Component 3 axis positively characterized by the PCB 194, canned haricot and canned fish (cos2 ≥ 0.7), and negatively by the PCB 149 (cos2 ≤  − 0.5), component 4 axis positively characterized by the PCB 149, and pasta source (cos2 ≥ 0.7). The components (1, 2, 3 and 4) translated which kinds of PCBs had a similar distribution in samples. As a result, the sample plot (Fig. 4) discriminated the correlation between the amount and type of PCBs in canned food.

## 4 Conclusion

For the first time, this research examined PCB residues in different canned foods in Tehran. The samples were examined by modified QuEChERS method and GC-MS technique that performed very well in this field. In conclusion, herbal food cans had higher concentrations of PCBs in comparison with canned fish, which this difference can be referred to the source of different types of PCBs and the food ingredients. By comparing the outcomes of this research with international standards, it was found the amount of this pollutant in canned food is completely less than the existing standard. The heat map and PCA demonstrated that the grouping of our different samples based on their PCBs profile was closely related and distinctly different in the canned samples. The MCS results displayed the cancer risk (ILCR) due to oral PCBs estimated in safe limit (CR > 1E-6). The PCBs exposure in both groups was under the tolerable daily intake (TDI < 10 ng kg^−1^ BW day^−1^). One of the limitations of this project is the lack of study on all the canned foods available in Iran, which was due to the limitation of financial resources. Finally, it is suggested to conduct research on other canned foods produced in Iran as well as imported products in the future.

## -

.

## CRediT authorship contribution statement

**Faezeh Vali Mohammadi:** Methodology, Formal analysis, Funding acquisition, Investigation, Software, Writing – original draft. **Peyman Qajarbeygi:** Visualization, Investigation, Methodology, Software, Validation, Project administration. **Nabi Shariatifar:** Conceptualization, Supervision, Methodology, Validation, Visualization, Writing – review & editing. **Razzagh Mahmoudi:** Methodology, Software, Validation, Data curation, Writing – original draft. **Majid Arabameri:** Methodology, Software, Validation, Data curation, Resources.

## Declaration of Competing Interest

The authors declare that they have no known competing financial interests or personal relationships that could have appeared to influence the work reported in this paper.

## Data Availability

No data was used for the research described in the article.
